# Relationship between destructive leadership styles and health workers’ mental health: a systematic review

**DOI:** 10.47626/1679-4435-2025-1378

**Published:** 2025-07-13

**Authors:** Magdalena Ahumada, Elisa Ansoleaga, Raúl Ramírez-Vielma, María José Mera-Lemp, Elena Soto-Contreras, Dennisse Brito-Placencia

**Affiliations:** 1 School of Psychology, Universidad Alberto Hurtado, Santiago, Chile; 2 School of Psychology, Universidad Diego Portales, Santiago, Chile; 3 Department of Psychology, Universidad de Concepción, Concepción, Chile; 4 Faculty of Juridical, Social and Educational Sciences, Universidad Viña del Mar, Viña del Mar, Chile; 5 School of Health and Dentistry, Universidad Diego Portales, Santiago, Chile; 6 Graduate Program in Psychology, Universidad Diego Portales, Santiago, Chile

**Keywords:** leadership, mental health, systematic review, health care personnel, liderazgo, salud mental, revisión sistemática, trabajadores de salud

## Abstract

Mental health has become a central topic, including its relationship with work. Health care workers are especially affected by the effects of work on mental health, with leadership emerging as a significant dimension for analysis. The aim of this study is to analyze the available evidence to understand the causes or antecedents of mental health problems among health care workers. A systematic review (International Prospective Register of Systematic Reviews CRD42022379794) was conducted, including empirical quantitative articles (experimental, quasi-experimental, and non-experimental) in English and Spanish that were related to the study variables. Theoretical and qualitative articles, systematic reviews, and meta-analyses were excluded. The data were analyzed through a narrative synthesis. Eighteen articles that presented various forms of destructive leadership were reviewed, such as abusive, toxic, *laissez-faire* leaderships and incivility behaviors. Destructive leadership styles were shown to increase the risk of mental health problems and affect workplace organizations, manifesting as absenteeism, turnover intention, and low satisfaction. The results of this literature review show the significant role of leadership in relation to mental health and, in particular, the negative effects of destructive leadership on health care workers.

## Abstract (pt)

La salud mental se ha consolidado como un tema central a nivel global, incluida su relación con el trabajo. Un sector altamente afectado por las repercusiones del trabajo en la salud mental es el de los trabajadores sanitarios, existiendo dimensiones relevantes para su análisis, como el liderazgo. El objetivo de este estudio es analizar la evidencia disponible para comprender las causas o antecedentes de los problemas de salud mental en los trabajadores sanitarios. Se realizó una revisión sistemática (International Prospective Register of Systematic Reviews, CRD42022379794), incluyendo artículos empíricos cuantitativos (experimentales, cuasiexperimentales, no experimentales), en inglés y español, vinculados con las variables del estudio. Se excluyeron artículos teóricos, cualitativos, revisiones sistemáticas y metaanálisis. La información fue analizada a través de una síntesis narrativa. Se revisaron 18 artículos que contenían distintas formas de manifestación de liderazgos destructivos: abusivo, tóxico, *laissez-faire* y conductas de incivismo. Se evidenció que estilos de liderazgos destructivos aumentan el riesgo de problemas de salud mental y afectan la organización del trabajo, manifestándose en ausentismo, intención de renunciar y baja satisfacción laboral. Los resultados encontrados en esta revisión de literatura evidencian el relevante papel del liderazgo en la salud mental, especialmente en cuanto a los efectos negativos que los liderazgos destructivos ejercen en los trabajadores de salud.

## INTRODUCTION

Mental health has emerged as a key global concern, due to both its associations and consequences for numerous social domains, including the work context. This issue is linked to workers’ well-being and quality of life, and its negative impact is reflected in decreased work effectiveness and efficacy. There is an estimated loss of 12 billion working days each year due to depression and anxiety, resulting in a cost of one trillion dollars to the global economy annually.^1^

In recent years, particularly in the context of the COVID-19 pandemic, health care has gained special relevance as a work setting where the effects of mental health are clearly evident.^2,3^ In this scenario, leadership styles seem to play a crucial role.^4^ However, previous studies on leadership in the health care field have mainly focused on its relationship with care quality, effectiveness. or innovation in health,^5-7^ while overlooking its association with workers’ health.

The relevance of health care work as a context is significantly related to transformations in labor activity, in which the service sector has gained a more prominent role.^8,9^ This population, in particular, is characterized by the fact that workers are both the providers and the deliverers of the service. Moreover, it is not always possible to plan a regular work pace, since services are provided on demand and may often involve unexpected tasks. Additionally, service quality depends on the worker-client/user relationship. It is also important to highlight that this relationship represents an additional demand for workers, often requiring “emotional labor,” a concept that refers to the need for workers to express the “right” emotions. Such emotions influence service quality and may differ from workers’ true emotions.^10^

An additional relevant aspect is that health care workers typically face complex work processes, high levels of uncertainty and autonomy, as well as high work intensity, due to long working hours and heavy workloads.^11^ Furthermore, the relational dimension is highlight valued, since it is considered a work tool that represents both a demand and a source of strain, also determining the quality of the worker-client/user relationship and setting the boundaries and possibilities for carrying out their duties.^8^ Another aspect to consider, consistent with global patterns, is the predominance of women in this labor sector, where gender inequalities among workers reflect those present in the overall labor market.^12^

When it comes to the critical role leadership can play in the health care field, it is worth noting that most of the theoretical framework on this matter have been developed in business environments and have only recently have been applied to health care settings,^13^ which present distinct contextual challenges.^5^ Ignoring the impact that leadership can have on the mental health of health care workers is not only objective from the ethical and legal standpoint, but also negatively affects the quality of care provided to service users.^2^

In fact, evidence suggests that leaders in health care setting may negatively affect workers’ mental health through harmful leadership practices, exposure to violence, or by neglecting aspects of work organization and failing to recognize how these aspects interact with social dimensions such as gender and social power imbalances, among others.^14^

Although mental health problems are multifactorial, strong evidence shows how certain working conditions and organizational structures negatively affect workers’ mental health.^15^ One the most significant dimensions of work related to workers’ mental health is workplace violence.^16-18^ Although these behaviors can occur downward, horizontally, or upward, the literature shows that approximately 80% of cases of workplace bullying happen in a top-down manner.^19^ Thus, leadership styles play a crucial role in understanding the occurrence of workplace violence.

It is important to emphasize that the study of destructive leadership has gained relevance over the past 15 years, notably in the reviews conducted by Schyns & Schilling,^20^ Mackey et al.,^21^ Einarsen et al.,^22^ among others. One of the most widely used conceptual models is that proposed by the latter authors regarding destructive leadership.^22^ This behavioral model posits that the leader may exhibit destructive behaviors in one domain while being constructive in another.^23^

Destructive leadership is defined as the systematic and repeated behavior by a leader, supervisor or manager that violates the legitimate interest of the organization by undermining and/or sabotaging the organization’s goals, tasks, resources, and effectiveness and/or the motivation, well-being and job satisfaction of their subordinates.^22^ Destructive leadership styles include: supportive-disloyal (pro-subordinates and anti-organization); derailed (anti-organization and anti-subordinates); tyrannical (pro-organization and anti-subordinates); and *laissez-faire* (the leader tries to avoid decision-making and responsibilities associated with their position)^22^. All of these leadership behaviors are harmful to both organizations and individuals. For instance, destructive leadership, especially *laissez-faire* and tyrannical styles, provides “fertile ground” for workplace violence.^20,22,24,25^

Additionally, other models have emphasized the importance of abusive supervision styles, characterized by hostile verbal and nonverbal behaviors,^26^ or aversive leadership, which relies on intimidation, coercion, and punitive actions.^27^ Other authors have proposed concepts such as despotic,^28^ narcisistic,^29^ or exploitative^30^ leadership to describe styles focused on achieving leaders’ personal interests.

Research on destructive leadership has documented its detrimental effects on subordinates.^24^ Empirical evidence shows that this type of leadership represents a persistent problem within organizations, given its negative impact on performance, absenteeism, turnover intention, job dissatisfaction, and staff turnover, among others.^26,31^ It has also been suggested that this leadership style negatively affects key workplace outcomes that are crucial to the effective functioning of the organization.^21,31^

Although some studies^31,32^ have examined the relationship between destructive leadership and mental health, this topic needs to be systematically explored in dept with regard to the available empirical evidence. Nevertheless, destructive leadership has proven to exert a substantial negative impact on workers’ mental health. Skakon et al.^33^ concluded that positive leadership is associated with workers’ well-being, whereas negative leadership is related to emotional exhaustion,^34,35^ poorer emotional health, and increased burnout.^36^

Moreover, Schyns & Schilling’s meta-analysis^20^ found a negative correlation between destructive leadership and followers’ positive outcomes and behaviors (eg, attitudes towards the leader, well-being, and individual performance) and positive correlations with negative outcomes(eg, turnover intention, resistance towards the leader, counterproductive work behavior). Additionally, Nielsen et al.^37,38^ reported that *laissez-faire* leadership and workplace bullying are the strongest predictors of subsequent distress, and that tyrannical leadership behaviors remain a significant stress factor even after controlling for the effects of bullying behaviors. Lastly, Lui et al.^32^ demonstrated that abusive supervision was significantly associated with suicidal ideation, with regression analysis results indicating that meaning in life both moderated and mediated the relationship between abusive supervision and suicidal ideation.

Considering the evidence presented regarding both the importance of health care work in mental health and the potential impact of destructive leadership in this field, it is crucial to understand how this phenomenon occurs among health care professionals. Therefore, the present study aims to answer the following question: how does scientific literature characterize the relationship entre destructive leadership styles and health care workers’ mental health? Furthermore, the study seeks to describe the available evidence to understand the underlying causes of mental health problems among health care workers, specifically destructive leadership.

## METHODS

The protocol for this systematic review was registered in the International Prospective Register of Systematic Reviews (PROSPERO) under registration number CRD42022379794.

### SEARCH STRATEGY

Published articles were retrieved from the following databases: PubMed, Web of Science, SciELO, and Scopus, written in both English and Spanish. Only studies from 2012 to 2022 were included.

The review included quantitative empirical studies – experimental; quasi-experimental, and non-experimental (cross-sectional and cohort) – that addressed the study variables. Theoretical articles, qualitative studies, systematic review, and meta-analyses were excluded.

### DATA COLLECTION

The Covidence online platform was used to manage all stages of the review. Duplicate records were identified and removed. In the initial phase, two reviewers examined the titles and abstracts of the studies selected. Subsequently, full-text articles were reviewed independently by two different reviewers, and any disagreements were resolved by a third reviewer. Finally, data extraction was performed by a single reviewer, and any disagreement was resolved by a second reviewer.

**Figure 1:**
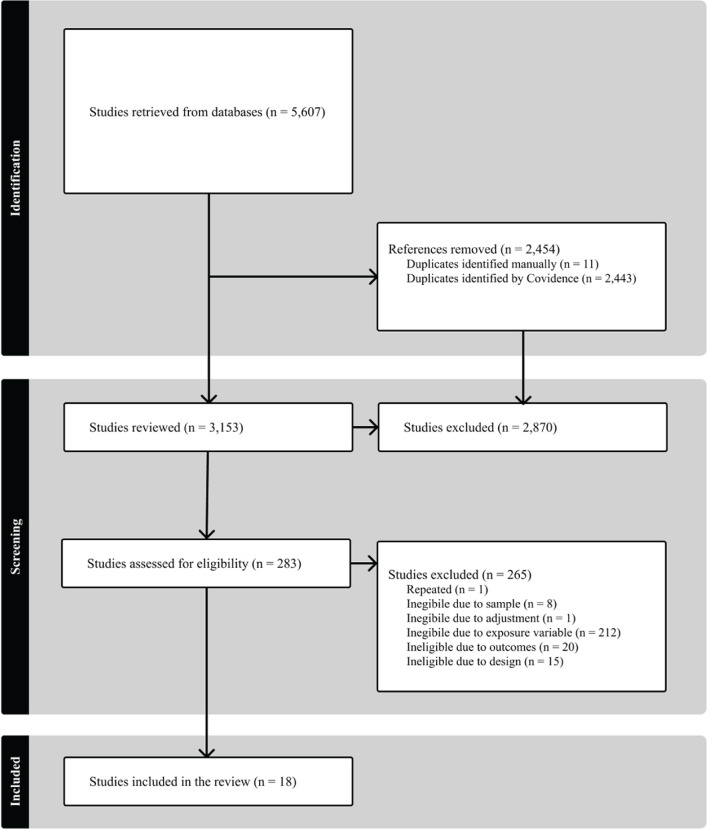
Summary of evidence review.

A total of 5,607 articles were retrieved from databases. After duplicates were removed, an initial screening of 3,153 articles was conducted based on title and abstract review in accordance with the inclusion criteria. Subsequently, 283 articles underwent full-text review, of which 265 were excluded for not meeting one or more review criteria, such as non-alignment with the exposure variable or outcome measures (further details in Figure 1). Finally, data extraction was conducted on the remaining 18 articles (Table 1).

**Table 1:** List of articles reviewed

Year	Authors	Title	Journal	DOI
2012	Leiter et al.	Getting better and staying better: assessing civility, incivility, distress, and job attitudes one year after a civility intervention	Journal of Occupational Health Psychology	http://doi.org/10.1037/a0029540
2013	Laschinger et al.	Workplace incivility and new graduate nurses’ mental health: the protective role of resiliency	Journal of Nursing Administration	http://doi.org/10.1097/NNA.0b013e31829d61c6
2013	Wing et al.	The influence of empowerment and incivility on the mental health of new graduate nurses	Journal of Nursing Management	http://doi.org/10.1111/jonm.12190
2014	Rodwell et al.	Abusive supervision and links to nurse intentions to quit	Journal of Nursing Scholarship	http://doi.org/10.1111/jnu.12089
2015	Read & Laschinger	Correlates of new graduate nurses’ experiences of workplace mistreatment	Journal of Nursing Administration	http://doi.org/10.1097/NNA.0b013e3182895a90
2015	Jeon et al.	Cluster Randomized Controlled Trial of An Aged Care Specific Leadership and Management Program to Improve Work Environment, Staff Turnover, and Care Quality	Journal of American Medical Director Association	http://doi.org/10.1016/j.jamda.2015.04.005.
2015	Qian et al.	Mental health risks among nurses under abusive supervision: the moderating roles of job role ambiguity and patients’ lack of reciprocity	International Journal of Mental Health Systems	http://doi.org/10.1186/s13033-015-0014-x
2016	Wood et al.	Managerial abuse and the process of absence among mental health staff	Work, Employment and Society	http://doi.org/10.1177/0950017015613755
2018	Abubakar	Linking work-family interference, workplace incivility, gender and psychological distress	Journal of Management Development	http://doi.org/doi.org/10.1108/JMD-06-2017-0207
2018	Mullen et al.	Destructive forms of leadership The effects of abusive supervision and incivility on employee health and safety	Leadership and Organization Development Journal	http://doi.org/10.1108/LODJ-06-2018-0203
2019	Booth et al.	Bad bosses and self-verification: The moderating role of core self-evaluations with trust in workplace management	Human Resource Management	http://doi.org/10.1002/hrm.21982
2020	Lee & Kim	Nursing stress factors affecting turnover intention among hospital nurses	International Journal of Nursing Practice	http://doi.org/10.1111/ijn.12819
2020	Sabbah et al.	The association of leadership styles and nurses well-being: a cross-sectional study in healthcare settings	The Pan African Medical Journal	http://doi.org/10.11604/pamj.2020.36.328.19720
2020	Labrague et al.	Influence of toxic and transformational leadership practices on nurses’ job satisfaction, job stress, absenteeism and turnover intention: A cross-sectional study	Journal of Nursing Management	http://doi.org/10.1111/jonm.13053
2020	Majeed & Fatima	Impact of exploitative leadership on psychological distress: A study of nurses	Journal of Nursing Management	http://doi.org/10.1111/jonm.13127
2022	Burr et al.	Factors associated with a positive view of respiratory care leadership	Respiratory Care	http://doi.org/doi.org/10.4187/respcare.10081
2022	Parent-Lamarche et al.	Going the Extra Mile (or Not): A Moderated Mediation Analysis of Job Resources, Abusive Leadership, Autonomous Motivation, and Extra-Role Performance	Administrative Sciences	http://doi.org/doi.org/10.3390/admsci12020054
2022	Zhang et al.	Does abusive supervision lead nurses to suffer from workplace violence? A cross-sectional study	Journal of Nursing Management	http://doi.org/10.1111/jonm.13326

### DATA ANALYSIS

Categories were established based on predefined variables used to guide data extraction from the selected literature (PROSPERO protocol). For this purpose, a database was created, including 12 categories that were used for both data extraction and to guide subsequent analysis (Table 2).

**Table 2:** Description of the categories

Category	Description
Author(s)	Main author’s name
Study title	Title of the selected article
Year	Year of publication
Country	Country where the study was conducted
Study design	Empirical studies employing quantitative methodology (experimental, quasi-experimental, or non-experimental).
Study site(s)	Number of study sites evaluated (one or more)
Study unit	Type of unit analyzed (eg, a specific unit or the entire hospital)
Sex	Binary sex (male-female)
Variables addressed in study objectives	Identification of the variables analyzed and proposed in the study objectives
Destructive leadership	Type of destructive leadership and scale used to measure it
Mental health variables	Mental health outcomes related to destructive leadership
Organizational outcomes	Organizational outcomes related to destructive leadership

Subsequently, a narrative synthesis^39,40^ was conducted to systematize the results obtained, with a main focus on the distribution of the categories for each of them.

### QUALITY ASSESSMENT

The risk of bias was assessed by two independent reviewers, using an adapted version of the Navigation Guide tool. The dimensions considered were: recruitment strategy; exposure assessment; outcome assessment; confounders; incomplete outcome data; selective outcome reporting; incompatibility; other sources of bias. Each dimension was graded according to the following options: “Low risk,” “Probably low risk,” “Probably high risk,” “High risk,” or “Not applicable.”

With regard to the quality assessment, the overall risk for all cases was “low” or “probably low.” It is worth noting that most of the reviewed articles were cross-sectional; therefore, questions such as “Was knowledge of the exposure adequately avoided during the study?” were classified as “Not applicable.”

Furthermore, most of the reviewed articles do not indicate their funding source (only five report this information). Some of the funding organizations for these articles include the Government of Wales, the University of Toronto, the Global Health Research Unit on Global Surgery, and the Basic Scientific Research Projects (No. 31041180139).

## RESULTS

The 18 selected articles were published from 2012 to 2022. Most of these studies were conducted in English-speaking countries such as Canada (27%), Australia (11%), England (9%) and the United States (9%)and. The remaining studies were conducted in, as well as in other countries such as China (11%), Pakistan, the Philippines, Nigeria, Korea, and Lebanon. None of the reviewed articles were published in Spanish-speaking countries.

In relation to study design, 89% (n = 16) were non-experimental, and the remaining 11% (n = 2) were experimental. Among the non-experimental studies, 72% (n = 13) were cross-sectional, 11% (n = 2) were cohort studies, and 6% (n = 1) assessed two studies simultaneous, of which one was cross-sectional and the other longitudinal. Among the experimental and quasi-experimental studies, 6% (n = 1) were randomized, whereas 6% (n = 1) were non-randomized.

With regard to the samples used, 10 of the articles included in the review were conducted in more than one center, whereas six were conducted on a single site, and two articles did not provide this information.

Furthermore, eight of these (36.4%) used hospital samples, and an equal number (n = 8; 36.4%) used samples from specific units. In contrast, three articles (13.6%) did not report the area or unit of their samples. Moreover, one study (4.5%) drew its sample from a health center, and another from a residential facility (n = 1; 4.5%). Finally, a single article (4.5%) included all the previously mentioned study sites – hospitals, specific units, residential facilities, and health centers.

All 18 sources (100%) included both sexes in their samples; however, no further gender-specific analyses were performed. In addition, 11 articles used nurses as their only sample, three included nursing staff alongside other health care workers such as doctors, administrative personnel, or health assistants. Finally, only three studies did not specify the type of health worker used in their sample, and one included exclusively health assistants and their supervisors.

Of the total number of studies reviewed, four (22%) addressed destructive leadership and mental health in their objective, four addressed leadership and organizational variables (22%), and five (28%) addressed violence and mental health. Moreover, five articles covered destructive leadership, mental health, and organizational variables (28%).

As for the type of destructive leadership evaluated, 28% were assessed using the concept of incivility, 33% using abusive leadership, 6% toxic leadership, and 11% *laissez-faire* leadership. The remaining articles employed other concepts, such as “exploitative leaders” or “supervisor social undermining.” In general, most of the articles used scales to evaluate the concept (n = 16), and only three used single-item questions. The three most frequently used scales were the Abusive Supervision Scale,^41^ Workplace Incivility Scale,^42^ and the Toxic Leadership Behaviors of Nurse Managers Scale^43^ (Table 3).

**Table 3:** Number of articles according to destructive leadership type, mental health outcomes, and organizational outcomes

	n	%	n_i_
Type of destructive leadership			
Incivility	5	28	0.28
Abusive leadership	6	33	0.33
Toxic leadership	1	6	0.06
*Laisse-faire* leadership	2	11	0.11
Other	4	22	0.22
Total	18	100	1.00
Mental health outcomes			
Burnout	2	11	0.11
Distress	9	50	0.50
Mental health	6	33	0.33
Depressive or anxious symptoms	1	6	0.06
Total	18	100	1.00
Organizational outcomes			
Job satisfaction	4	27	0.27
Absenteeism	2	13	0.13
Turnover intention	2	13	0.13
Job turnover	4	27	0.27
Other	3	20	0.20
Total	15	100	1.00

n_i =_ relative frequency.

In relation to the forms of measuring mental health, half of the studies employed measures related to distress, while 33% were used mental health scales such as the General Health Questionnaire^44^ or the 36-Item Short Form Health Survey.^45^ Finally, only two articles assessed burnout using questionnaires such as the Maslach Burnout Inventory-General Survey,^46^ and another evaluated depressive or anxious symptoms with the Kessler-10 questionnaire.^47^

All articles reported negative effects of destructive leadership on mental health. For instance, individuals with higher scores in abusive supervision and role ambiguity were at greater risk for mental health (b = 0,40; standard error [SE] = 0,06; BCa 95%CI = 0,2826-0,5093).^48^ Furthermore, Labrague et al.^49^ found that toxic leadership behaviors predicted workplace distress (β = 2.63; p < 0.01), and Read & Laschinger^50^ observed that poor mental health was associated with high levels of leadership incivility (Pearson r 0.28).

Conversely, all of the 10 articles that included organizational outcomes measured more than one, with the majority involving job satisfaction (27%) and job turnover (27%). Absenteeism (13%) and turnover intention (13%) were less frequently evaluated.

The results obtained indicated that toxic leadership behaviors predicted absenteeism (β = 0.17; p < 0.001), turnover intention (β = 0.22; p < 0.001), and job satisfaction (β = -0.10; p < 0.01).^49^ With regard to the latter variable, Read & Laschinger^50^ found that it was related to supervisor incivility (Pearson r = -0.24) and job turnover (Pearson r = 0.19). Finally, Burr et al.^51^ reported the association between negative leadership and missing work for any reason (category: other) (0.69 [95%CI 0.90-0.99]).

## DISCUSSION

The aim of this article was to describe the available evidence to better understand the causes or antecedents of mental health problems among health care workers, with a focus on destructive leadership. Eighteen articles that documented different forms of destructive leadership were reviewed, among which the following categories were predominantly identified: abusive, toxic, *laissez-faire* leadership, and leader incivility.

Exposure to destructive leadership was found to bring consequences for health care workers’ mental health, especially in relation to outcomes such as burnout or distress. This is consistent with the available literature, showing that health care leaders can negatively affect workers’ mental health through destructive leadership.^14,24,36^ Additionally, one of the reviewed articles^50^ discussed the relevance of leadership styles in understanding the occurrence of workplace violence, which aligns with Zapf & Einarsen’s findings^19^ on the high prevalence of downward workplace bullying and its relevance in the study of leadership behaviors.

Likewise, a review of the results related to destructive leadership and organizational dimensions found that destructive leadership had a negative effect on absenteeism, turnover intention, and job satisfaction among health care workers. This in consistent with the literature on the general workforce, which reports that there are adverse effects on organizational variables, along with a detrimental impact on performance.^26,31^ However, it is evident that leadership style plays a significant role in organizational outcomes (ie, variables essential for the effective functioning of the organization^21,31^) and that, in the health care sector, this may also negatively impact service delivery to pacients.^2^

In conclusion, while much of the scientific literature focuses on the importance of leadership in health care due to its impact on quality of care and management,^52,53^ the findings from this literature review highlight the significant role of leadership in relation to mental health, and particularly the negative effects of destructive leadership on health care workers. Even though our findings contribute to advancing the study of health care workers’ health, especially in the Latin American context, where there is already a systematic body of evidence on workplace violence^54^ and/or health care workers’ mental health,^55,56^ the relationship between these issues and leadership practices remains an underexplored area of research.

This article presents limitations related, in part, to the inclusion of articles conducted during the COVID-19 pandemic, when mental health outcomes may have been influenced by the effects of working under emergency conditions. Nevertheless, studies conducted during the pandemic showed that appropriate leadership can serve as a protective factor for workers’ mental health.^2^ Another limitation lies in the fact that only articles in Spanish and English were included, thereby excluding evidence published in other languages. Future studies should incorporate evidence from other languages in order to expand the coverage of the review, which may also imply greater cultural diversity in the phenomenon under study.
